# Editorial: Microbiomes of art and their importance in preserving cultural heritage

**DOI:** 10.3389/fmicb.2023.1321133

**Published:** 2023-11-10

**Authors:** António Portugal, Xiaobo Liu, Adam Pyzik, João Trovão

**Affiliations:** ^1^Department of Life Sciences, Centre for Functional Ecology (CFE) – Science for People and the Planet, University of Coimbra, Calçada Martim de Freitas, Coimbra, Portugal; ^2^FitoLab – Laboratory for Phytopathology, Instituto Pedro Nunes (IPN), Coimbra, Portugal; ^3^TERRA – Associate Laboratory for Sustainable Land Use and Ecosystem Services, Department of Life Sciences, University of Coimbra, Coimbra, Portugal; ^4^School of Environmental and Biological Engineering, Nanjing University of Science and Technology, Nanjing, Jiangsu, China; ^5^Department of Building Materials Engineering and Geoengineering, Faculty of Civil Engineering and Architecture, Lublin University of Technology, Lublin, Poland; ^6^Department of Environmental Microbiology and Biotechnology, Faculty of Biology, Institute of Microbiology, University of Warsaw, Warsaw, Poland

**Keywords:** microbiome, cultural heritage, art, biodeterioration, conservation, restoration, archaeological wood, biocides

Over the past decade, there has been a growing interest surrounding the “*Microbiomes of art and their importance in preserving cultural heritage*.” Advances in science and technology have led to the development of several innovative strategies in the restoration and conservation of artwork and cultural heritage. These advances have been fostered by improvements in the research techniques that enable us to gather insights from different approaches, together with the need to better understand the field in order to preserve the cultural and historic significance of art in our society for future generations. However, there are still several challenges within the field, with a notable example being the preservation of ancient rock and cave paintings, and also waterlogged archaeological wood, to which bacteria, archaea, fungi and microalgae pose a great threat due to the inability to regulate the microclimate as can be done in museums and galleries. Thus, it is evident that further enhancing our understanding of the dynamics between microorganisms and the art they inhabit will be essential in future approaches to the preservation and restoration of artwork.

Whilst the community can be proud of recent advances, from the application of probiotics to paintings in order to prevent biodeterioration, to the potential of microbiome analysis in the detection of forgeries, there is still a long way to go. In this Research Topic, we encouraged researchers to explore the recent developments and main challenges faced by the field. We also aimed to get contributions that would investigate the future and define the directions that the field would take in the coming years, giving answers to some of the following questions: What have been the key discoveries so far? What are the most pressing matters that need to be addressed? Where is research going to take us in the forthcoming years?

Through this article collection, we tried to explore key aspects of “*Microbiomes of art and their importance in preserving cultural heritage*.” From this, we aimed to facilitate the dissemination of knowledge in order to encourage the development of innovative solutions to the challenges faced by the field today.

We gathered six important published papers (four original research articles, one review and one methods article) that gave responses to several of the subtopics and questions that we aimed to be explored in this Topic:

1) Characterization of the diverse microbiomes of artworks, including archaeological wood, paintings, murals, monuments, and cultural heritage sites.
2) Developing or revisiting modern and innovative tools and protocols aimed toward the prevention and conservation of art biodeterioration, as well as contributions detailing conservation and restoration treatments.
3) Enhancing our understanding of microbial threats to the preservation of art and the cultural assets.

The review by Geweely gathered important information about the recent conservation techniques of organic and inorganic archaeological artifacts against microbial deterioration. An outline of comparative new protective methods for conserving plant-origin organic artifacts [fibers (manuscripts and textiles) and wood], animal-origin organic artifacts (paintings, parchments and mummies) and inorganic stone artifacts were investigated. The work not only definitely contributes to the development of safe revolutionary ways for more efficient safe conservation of items of historical and cultural worth but also serves as a significant diagnostic signature for detecting the sorts of microbial identification and incidents in antiques.

In the methods article by Flocco et al., the authors review the main experimental challenges and propose a standardized workflow (step-by-step overview) to study the microbiome of cultural heritage objects, illustrated by the exploration of bacterial taxa. The methodology was developed targeting the challenging side of the spectrum of cultural heritage objects, such as the delicate written records, while retaining flexibility to adapt and/or upscale it to heritage artifacts of a more robust constitution or larger dimensions, aiming to facilitate the interdisciplinary investigation and interactions among the cultural heritage research community.

The very fitting and original research article by Beccaccioli et al. deals with the evaluation of biological degradation of waterlogged archaeological wood, which is crucial for choosing the right conservative and protective strategies. In this fine paper, the microbial communities were evaluated through the sequencing of ITS sequences for fungi and 16S rRNA gene sequences for bacteria using the portable Oxford Nanopore Technologies (ONT) MinION platform enabling rapid real-time sequencing.

In another interesting original research article by Liu et al., the authors collected 33 samples, including 27 aerosol and 6 mural painting samples, from different sites of Xu Xianxiu's Tomb, and analyzed them using culture-dependent methods. The authors compared the diversity of culturable bacteria and fungi isolated from the air and murals, and explored the potential impacts of those microorganisms on the biodeterioration of the murals.

Zalar et al. offered us a very exciting original research paper in which they aimed to identify the cultivable fungal diversity in several canvas paintings from different origins and utilize the gathered information to create prediction models, using machine learning methods, for the occurrence of certain taxa concerning the constituent materials of the canvas paintings and potential damages that may occur due to their presence. The data obtained holds significant potential for being a highly interesting and citable study with particular relevance for the preservation of these historical assets.

Finally, we got a very thrilling original research article, authored by Bartoli et al. that explored the close relationship between Science and Art, colonization of artworks by microorganisms, and developing new technologies that can be used to support the creation and durability of bio-artworks. In this sense, the authors performed *in situ* testing of several biocides and water repellents and monitored their efficacy to eventually increase the durability of graphical artworks.

We hope that these diverse articles can be read and spread by researchers, stakeholders and technicians and that the findings brought to light can be translated into new conservation and restoration actions in art objects, aiming at preserving our Cultural Heritage. This was our main goal when we accepted carrying out this mission with enthusiasm and scientific rigor!

## Author contributions

APo: Conceptualization, Writing—original draft, Writing—review & editing, Validation. XL: Writing—review & editing, Validation. APy: Writing—review & editing, Validation. JT: Writing—review & editing, Validation.

